# Evaluation of Anxiolytic Activity of Angiotensin Receptor Blockers Using Actophotometer Test in Wistar Rats

**DOI:** 10.7759/cureus.69798

**Published:** 2024-09-20

**Authors:** Chavda Akshayraj Vanrajbhai, Vandana M Thorat, Vedant S Patel, Kartiki P Patil, Leesha L Chawla

**Affiliations:** 1 Department of Pharmacology, Krishna Institute of Medical Sciences, Krishna Vishwa Vidyapeeth (Deemed to be University), Karad, IND

**Keywords:** actophotometer, angiotensin receptor blockers, anxiolytic, diazepam, locomotor activity, losartan, telmisartan, wistar rats

## Abstract

Introduction

Anxiety disorders are common mental illnesses impacting quality of life, with current treatments like benzodiazepines and selective serotonin reuptake inhibitors (SSRIs) facing limitations due to side effects. Angiotensin receptor blockers (ARBs), used primarily for hypertension, have shown potential neuropsychiatric benefits, including anxiolytic effects. This study explores the anxiolytic effects of two ARBs, telmisartan and losartan, by evaluating locomotor activity in Wistar rats, aiming to identify new treatment options for anxiety through modulation of the renin-angiotensin system.

Aim

To evaluate the anxiolytic activity of telmisartan and losartan in experimental Wistar rats.

Materials and methods

The study was carried out after consent was obtained from the Institutional Animal Ethics Committee (IAEC). The rats were divided into four groups: group 1 (control group) received distilled water (2 ml/kg), group 2 (diazepam 2 mg/kg group) received diazepam (2 mg/kg), group 3 (telmisartan 5 mg/kg group) received telmisartan (5 mg/kg), and group 4 (losartan 5 mg/kg group) received losartan (5 mg/kg). The actophotometer test, which measures the locomotor activity levels of rats, was utilized to evaluate their anxiolytic activity. The percent decrease in locomotor activity was calculated for statistical evaluation.

Results

Our investigation found that the telmisartan 5 mg/kg and losartan 5 mg/kg groups had considerable anxiolytic activity (p<0.05) compared to the control group, and it was comparable to the diazepam 2 mg/kg (p>0.05) group.

Conclusion

The findings of our study indicate that ARBs, specifically telmisartan 5 mg/kg and losartan 5 mg/kg, exhibit potential anxiolytic effects, evidenced by a significant reduction in locomotor activity in the actophotometer test. These results imply that ARBs could be considered as possible therapeutic agents for anxiety, providing a new perspective on their use beyond traditional cardiovascular applications.

## Introduction

Anxiety disorders are a significant public health concern in India, affecting millions of individuals and contributing substantially to the overall burden of mental disorders in the country. According to the Global Burden of Disease Study 1990-2017, an estimated 44.9 million people (with a 95% uncertainty interval (UI) of 41.2-48.9 million) in India were living with anxiety disorders in 2017. This highlights the substantial impact of anxiety disorders on the population. Furthermore, anxiety disorders accounted for 19.0% (15.9-22.4%) of the total disability-adjusted life years (DALYs) associated with mental disorders in India in 2017, underscoring their role as a significant contributor to the nation's mental health burden. Addressing anxiety disorders is crucial for reducing this burden and improving overall mental health outcomes in India [1]. Current pharmacological therapies, such as benzodiazepines and selective serotonin reuptake inhibitors (SSRIs), while beneficial, frequently have limitations, including adverse effects and insufficient efficacy [2]. As a result, there is an urgent need for innovative therapeutic agents with superior efficacy and safety profiles.

Angiotensin receptor blockers (ARBs) are drugs that primarily treat hypertension and heart failure by inhibiting the effects of angiotensin II at the angiotensin II type 1 receptor (AT1R). Recently, there has been a renewed interest in ARBs' possible neuropsychiatric advantages, particularly their anxiolytic qualities. Beyond its well-known involvement in cardiovascular control, the renin-angiotensin system (RAS) has been linked to a variety of brain activities, including neuroinflammation, oxidative stress, and neural plasticity [3].

Research indicates that ARBs like losartan possess anxiety-reducing properties in preclinical models. These effects are believed to stem from the drug's influence on AT1R. Investigations involving rodent models of anxiety demonstrate that losartan can alleviate anxiety-like symptoms, suggesting its potential as a therapeutic option for anxiety disorders. This anxiolytic effect is thought to be related to the modulation of the central RAS, which plays a crucial role in managing stress and anxiety responses​​ [4-6].

Similarly, telmisartan, another ARB, has shown promise in animal experiments in lowering anxiety-like behaviors, implying that ARBs may exert their effects via a variety of mechanisms, including regulation of neurotransmitter systems such as gamma-aminobutyric acid (GABA) [7-9].

The actophotometer test is a commonly employed behavioral assay to measure spontaneous locomotor activity in rodents. This test is particularly useful for assessing the impact of potential anxiolytic drugs on the central nervous system (CNS). Typically, a reduction in locomotor activity during the actophotometer test is interpreted as an anxiolytic-like response, suggesting a decrease in anxiety-related hyperactivity [10-12].

The purpose of this study is to examine the anxiolytic activity of two ARBs, telmisartan and losartan, in Wistar rats using the actophotometer test. By examining these compounds, we hope to shed light on ARBs' potential as innovative therapy alternatives for anxiety disorders as well as to gain a better understanding of the neuropsychiatric effects of RAS regulation.

## Materials and methods

Experimental animals

The experiment involved male and female Wistar rats weighing 150-300 grams, sourced from the central animal house at Krishna Vishwa Vidyapeeth (Deemed to be University) in Karad. A total of 24 Wistar rats, aged six to eight months, were divided into four groups, with six rats in each group, using a convenient sampling method. They were housed under standard environmental conditions and had unrestricted access to food and water until the experiment began. The rats were acclimatized to the laboratory environment at least thirty minutes before the experiment. All procedures adhered to the guidelines of the Committee for Control and Supervision of Experiments on Animals (CCSEA), and the study received approval from the Institutional Animal Ethics Committee (IAEC) under approval number KIMSDU/IAEC/02/2022.

Facilities and equipment

We got our rats from the central animal home at Krishna Vishwa Vidyapeeth (Deemed to be University), Karad. Various instruments were used for the experiment, such as a digital animal weighing machine, syringes, needles, glass beakers, a digital actophotometer (INCO, India), a stopwatch, and acrylic rat cages.

Drug administration and experimental protocol

The rats were divided into four groups: Group 1 (control group) received distilled water (2 ml/kg), group 2 (diazepam 2 mg/kg group) received diazepam (2 mg/kg), group 3 (telmisartan 5 mg/kg group) received telmisartan (5 mg/kg), and group 4 (losartan 5 mg/kg group) received losartan (5 mg/kg). A volume of 2 ml/kg of body weight of the medication was intraperitoneally supplied to each rat in accordance with their unique group assignment. A standard formula was used to convert the human clinical doses of all drugs used in the study to equivalent doses for rats. On the day of the experiment, all the drugs were freshly prepared and administered to the rats according to their respective groups.

Experimental method

The experimental procedure followed the steps outlined by Dews (1953) [13] and included the use of an actophotometer to measure the locomotor behavior of the animals. The animal was moved around in a square arena of the actophotometer. The actophotometer, which uses photoelectric cells linked in a circuit with a counter, makes it easy to monitor locomotor activity, also known as horizontal activity. When the animal blocked the light beam that would otherwise reach the photocell, a tally was made. Anxiolytic medication action was suggested by a low activity score, which indicated central nervous system depression, and central nervous system stimulation was indicated by an elevated activity score [14].

Procedure

The experiment was conducted by dividing the rats into four groups, each consisting of six rats (n=6). To begin, the rats were allowed to habituate to their environment by being placed individually in test cages for 30 minutes prior to the experiment. Following habituation, the basal locomotor activity of each rat was measured by placing them individually in the actophotometer for five minutes. After recording the baseline measurements, the rats were administered their respective drugs via intraperitoneal injection according to their group assignments. Thirty minutes post-drug administration, the locomotor activity of each rat was measured again by placing them individually in the actophotometer for five minutes to assess the effect of the administered drugs.

Evaluation

The locomotor activity score before and after treatment was noted, and the percent decrease in locomotor activity was calculated:

% decrease in locomotor activity = 1 - (A/B) × 100, 

where A = average locomotor activity score after drug administration, and B = average locomotor activity score before drug administration. 

Statistical analysis was conducted using one-way analysis of variance (ANOVA) to compare differences between groups, followed by the post hoc Tukey-Kramer multiple comparison test to identify specific group differences. Data were expressed as mean ± standard deviation (SD), and a p-value of less than 0.05 was considered statistically significant.

## Results

The results of the actophotometer test showing the locomotor activity scores before and after treatment in different groups and their comparisons are given in Table 1. The data are expressed as mean ± SD, with n=6 in each group.

**Table 1:** Effect of diazepam, telmisartan, and losartan pretreatments on locomotor activity in the actophotometer test in rats Before treatment (mean ± SD): Mean and SD of locomotor activity scores before treatment; After treatment (mean ± SD): Mean and SD of locomotor activity scores after treatment; Paired t-test: Statistical test used to compare before and after treatment scores within each group; t-value: Test statistic from the paired t-test; p-value: Probability value indicating the significance of the paired t-test results; One-way ANOVA with post hoc Tukey-Kramer test: Statistical test used to compare the means of multiple groups; F: Test statistic from the ANOVA; P: Probability value indicating the significance of the ANOVA results; A: Indicates significant difference when compared with the control group (p<0.0001). ANOVA: Analysis of variance; SD: Standard deviation

Group	Locomotor activity score		Paired t-test		One-way ANOVA with post hoc Tukey-Kramer test	
	Before treatment (mean ± SD)	After treatment (mean ± SD)	t-value	p-value	Before treatment	After treatment
Control	211 ± 09.96	209.67 ± 10.67	0.2020	0.8479	F=0.5666	F=135.16
Diazepam 2 mg/kg	217 ± 10.41	107.67 ± 11.71 ^A^	16.862	<0.0001		
Telmisartan 5 mg/kg	210 ± 12.02	113.5 ± 10.23^ A^	27.204	<0.0001	P=0.6434	P<0.0001
Losartan 5 mg/kg	217 ± 15.86	108.5 ± 9.35 ^A^	18.344	<0.0001		

Before treatment, the one-way ANOVA test produced a p-value of 0.6434, which is not considered significant. This suggests that the variation in group means was not significantly greater than what would be predicted by chance. After treatment, the one-way ANOVA test yielded a p-value of less than 0.0001, indicating high significance. This shows that the variation in group averages was much more than what would be predicted by chance (Table 1).

Post hoc analysis using the Tukey-Kramer multiple comparison test revealed that when compared with the control group, the diazepam 2 mg/kg, telmisartan 5 mg/kg, and losartan 5 mg/kg groups showed a statistically significant decrease in the mean locomotor activity score (p<0.05). This is represented in Table 1 as ‘A’. Furthermore, when compared with the diazepam 2 mg/kg group, telmisartan 5 mg/kg and losartan 5 mg/kg groups demonstrated similar effects on the mean locomotor activity score (p>0.05). Additionally, the losartan 5 mg/kg group showed similar effects to the telmisartan 5 mg/kg group on the mean locomotor activity score (p>0.05).

A paired t-test comparing locomotor activity scores before and after treatment revealed an extremely significant decrease in the mean locomotor activity score in the diazepam 2 mg/kg, telmisartan 5 mg/kg, and losartan 5 mg/kg groups (p<0.0001) after treatment compared to their respective pretreatment values.

**Figure 1:**
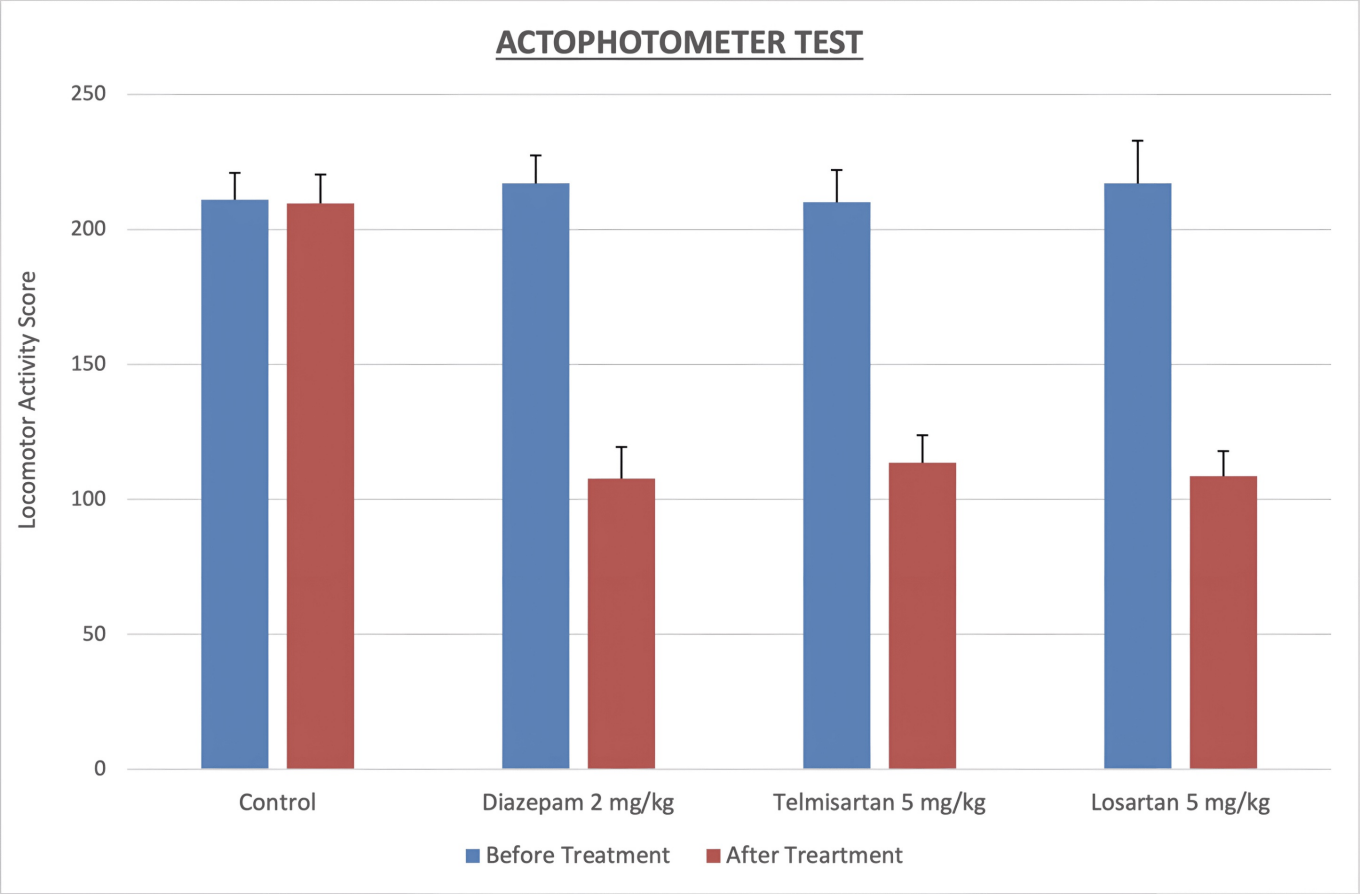
Effect of diazepam, telmisartan, and losartan pretreatments on locomotor activity in the actophotometer test in rats Values are shown as mean ± SD. SD: Standard deviation

The graphical representation of the actophotometer test results showing locomotor activity scores before and after treatment in different groups is presented in Figure 1.

The results of the actophotometer test showing the percent decrease in locomotor activity after treatment in different groups are presented in Table 2. The control group showed a minimal decrease of 0.63%, while the diazepam 2 mg/kg, telmisartan 5 mg/kg, and losartan 5 mg/kg groups exhibited substantial decreases of 50.38%, 45.95%, and 50%, respectively.

**Table 2:** Percent decrease in the locomotor activity in diazepam, telmisartan, and losartan pretreatment groups Before treatment (mean): Average locomotor activity score before treatment; After treatment (mean): Average locomotor activity score after treatment. % decrease in locomotor activity was calculated using the formula: (1 - (A/B)) × 100, where A = average locomotor activity score after drug administration, and B = average locomotor activity score before drug administration.

Group	Locomotor activity score		% decrease in locomotor activity
	Before treatment (mean)	After treatment (mean)	
Control	211	209.67	0.63%
Diazepam 2 mg/kg	217	107.67	50.38%
Telmisartan 5 mg/kg	210	113.5	45.95%
Losartan 5 mg/kg	217	108.5	50%

**Figure 2:**
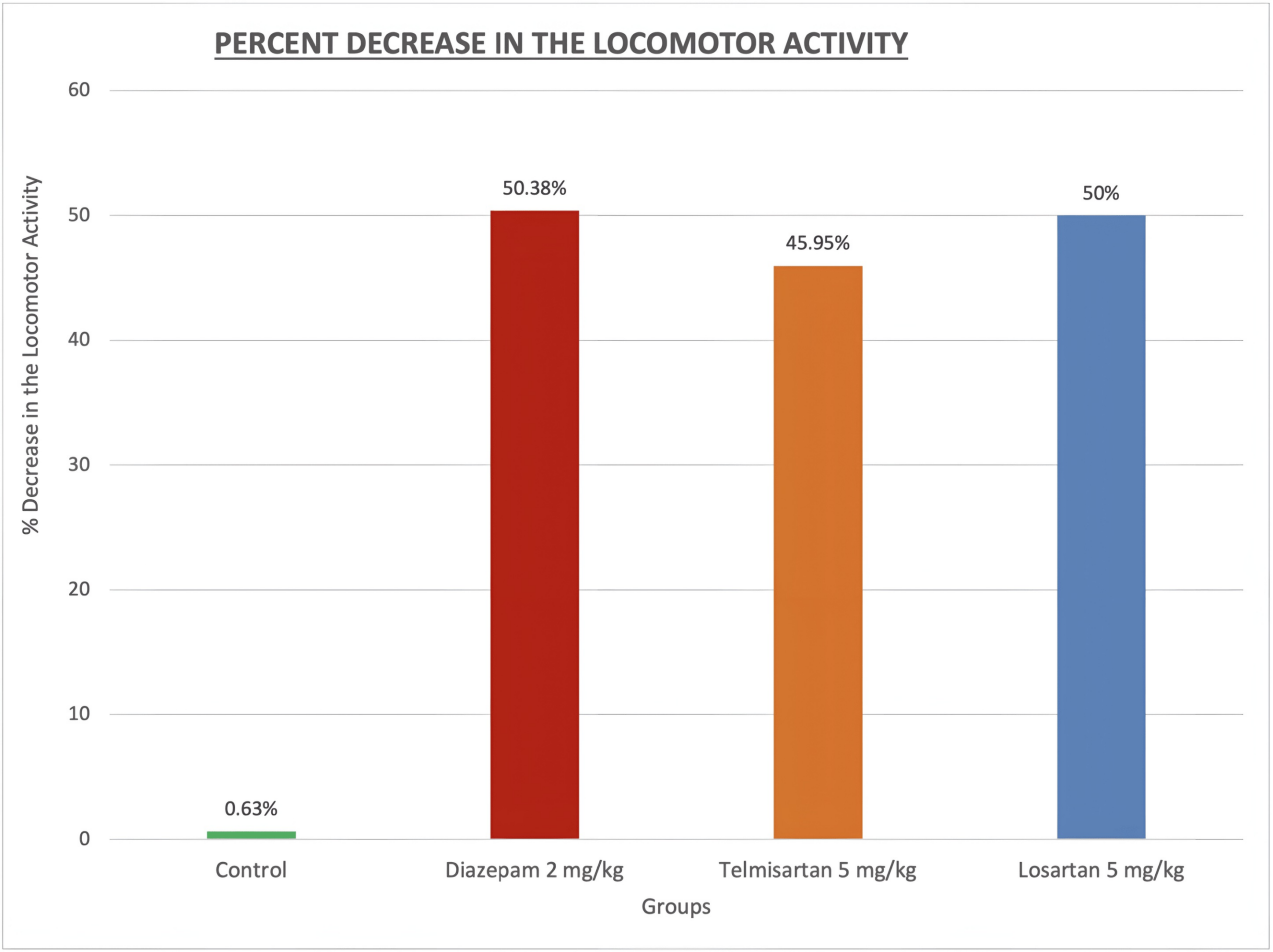
Percent decrease in the locomotor activity in diazepam, telmisartan, and losartan pretreatment groups Values are shown as percentages.

The graphical representation of these results illustrating the percent decrease in locomotor activity scores after treatment across the different groups is provided in Figure 2.

## Discussion

The rats were placed individually in the actophotometer chamber for five minutes and their basal locomotor activity was measured. The locomotor activity score was then collected for another five minutes, 30 minutes after the relevant therapy was administered. A significant decrease in mean locomotor activity score after treatment compared to before treatment indicated the presence of an anxiolytic effect in the medication. Before treatment, there was no significant variation among group means (p>0.05). After treatment, there was extremely significant variation among group means (p<0.0001) (Table 1).

Treatment with diazepam 2 mg/kg, telmisartan 5 mg/kg, and losartan 5 mg/kg resulted in a substantial decrease in mean locomotor activity score compared to the control group (p<0.05) (Table 1). When compared to the diazepam 2 mg/kg group, the telmisartan 5 mg/kg and losartan 5 mg/kg groups showed no significant difference in the mean locomotor activity score (p>0.05) (Table 1), indicating that the anxiolytic activity of both telmisartan 5 mg/kg and losartan 5 mg/kg is similar to diazepam 2 mg/kg.

Paired t-tests revealed that there was an extremely significant decrease in the mean locomotor activity score in diazepam 2 mg/kg, telmisartan 5 mg/kg, and losartan 5 mg/kg groups (p<0.0001) when compared to before treatment values.

The percent decrease in locomotor activity after the treatment indicates that diazepam 2 mg/kg, telmisartan 5 mg/kg, and losartan 5 mg/kg showed a significant decrease in locomotor activity (Table 2). Therefore, we can conclude that telmisartan 5 mg/kg and losartan 5 mg/kg do possess significant anxiolytic activity.

Our observations from the actophotometer test coincide with those of Srinivasan et al., who stated that losartan at doses of 5 mg/kg and 10 mg/kg significantly reduces anxiety in rats using the open field exploratory behavior test, social interaction test, and elevated plus maze test [15]. A study by Swetha et al. also concluded that ARBs such as losartan, olmesartan, and telmisartan have significant anti-anxiety properties at doses of 5 mg/kg and 10 mg/kg using the social interaction test and elevated plus maze test in rats [16].

Possible mechanisms behind telmisartan's anxiolytic activity

Telmisartan's anxiolytic activity may be beneficial for both stress-induced anxiety and generalized anxiety. By modulating the RAS, influencing neurotransmitter systems, and reducing oxidative stress, telmisartan can have broad applications in managing anxiety, regardless of its origin.

Modulation of RAS

Angiotensin II, through AT1R, can promote the release of stress hormones and increase anxiety-like behaviors. By blocking these receptors, telmisartan can reduce the influence of angiotensin II on stress and anxiety responses [17].

*Peroxisome Proliferator-Activated Receptor Gamma* (*PPAR-γ) Agonism*

Telmisartan is also a partial agonist of PPAR-γ. PPAR-γ activation can modulate neurotransmitter systems and reduce oxidative stress, inflammation, and neurodegeneration, all of which can affect anxiety levels [18].

Neurotransmitter Modulation

Telmisartan may influence neurotransmitter systems, including serotonin, dopamine, and GABAergic systems, by modulating receptor activity and intracellular signaling pathways. These systems play critical roles in mood regulation and anxiety. Telmisartan’s effect on these pathways may enhance serotonin and GABAergic activity, which promote anxiolytic effects, while reducing overactivity in dopaminergic circuits associated with stress and anxiety [19].

Telmisartan’s antioxidant properties may contribute to its anxiolytic activity by reducing oxidative stress, which plays a role in anxiety disorders. Oxidative stress can damage neurons and disrupt neurotransmitter systems involved in anxiety, such as GABA and serotonin. Telmisartan’s ability to lower oxidative stress may help preserve neuronal function, thus reducing anxiety symptoms. Studies have demonstrated telmisartan’s antioxidant effects in human endothelial cells, highlighting its potential neuroprotective benefits in the brain [20]. Additionally, antioxidants like those in *Oxalis latifolia* have been shown to have neuroprotective roles, supporting the idea that reducing oxidative stress can benefit neurodegenerative conditions, including anxiety [21]. Research on hypertensive rats showed that telmisartan reduced oxidative stress and improved peroxiredoxin-2 (Prdx2) expression, further supporting its potential to manage anxiety via antioxidant pathways [22]. 

Strengths and limitations of the study

The study has several strengths, particularly in exploring the novel anxiolytic effects of telmisartan and losartan, which are primarily known for their anti-hypertensive properties. This opens up the possibility of using these drugs for patients with comorbid conditions like anxiety, potentially reducing the need for multiple medications. Additionally, the study contributes to the growing body of research that links cardiovascular and neuropsychiatric health, suggesting a broader application of these drugs across different medical fields. However, the study also has a few limitations that need to be addressed. The anxiolytic activity of telmisartan and losartan was examined as secondary to their anti-hypertensive effects, and the effects of different doses were not assessed, leaving gaps in understanding their dose-response relationship. Moreover, there was no attempt to investigate the underlying mechanisms of their anxiolytic activity. Addressing these limitations in future studies will be crucial for a more comprehensive understanding of these drugs.

Implications of the study

The findings suggest that telmisartan and losartan may have therapeutic benefits beyond blood pressure control, particularly in anxiety management. If future research confirms these anxiolytic effects, it could lead to new guidelines for treating patients with both hypertension and anxiety disorders. Furthermore, understanding these drugs' impact on the brain's stress-related pathways might offer insights into how RAS interacts with anxiety regulation, expanding the therapeutic landscape for anxiety disorders.

Future direction

To address the study's limitations and expand upon its findings, future research should explore the effects of varying doses of telmisartan and losartan on anxiolytic activity, which could offer a more detailed understanding of their dose-response relationship. Additionally, investigating the mechanisms behind these drugs' anxiolytic effects will be important for uncovering how they influence anxiety pathways. Methods such as neuroimaging can be employed to observe changes in brain activity, while biomarker analysis may assess changes in stress-related hormones and neurotransmitters. Animal models of anxiety, such as the elevated plus maze or open field test, could also be used to further investigate their behavioral effects. Moreover, examining interactions with neurotransmitter systems like GABA or serotonin may provide deeper insights into their anxiolytic potential.

## Conclusions

Based on the outcomes of this experimental study, we conclude that telmisartan 5 mg/kg and losartan 5 mg/kg have anxiolytic activity comparable to the conventional anxiolytic medication diazepam 2 mg/kg. Cardiovascular problems can have an impact on mental health, particularly anxiety disorders, which are frequently associated with these chronic ailments. The current study reveals that using telmisartan and losartan in conjunction with conventional drugs may have an additional advantage in individuals with cardiovascular diseases such as hypertension and congestive heart failure associated with various anxiety disorders. In addition, pre-clinical evaluation and human studies are necessary to reinforce the results and demonstrate their usefulness in the long-term usage of telmisartan and losartan as possible anxiolytic agents in routine clinical procedures.

As a result, we propose that telmisartan and losartan, with their anti-hypertensive and extra-anxiolytic properties, may be more appropriate for individuals with pathological conditions that include hypertension, congestive heart failure, and anxiety disorders. Some examples include generalized anxiety disorder, panic disorder with or without agoraphobia, particular phobias, social anxiety disorder, separation anxiety disorder, and selective mutism. It is also worth noting that chronic illnesses such as hypertension and congestive heart failure can lead to a variety of anxiety disorders, with telmisartan and losartan acting primarily as anti-hypertensives and then producing anxiolytic effects.

## References

[1] (2020). The burden of mental disorders across the states of India: the Global Burden of Disease Study 1990-2017. Lancet Psychiatry.

[2] Baldwin DS, Anderson IM, Nutt DJ (2014). Evidence-based pharmacological treatment of anxiety disorders, post-traumatic stress disorder and obsessive-compulsive disorder: a revision of the 2005 guidelines from the British Association for Psychopharmacology. J Psychopharmacol.

[3] Wright JW, Harding JW (2013). The brain renin-angiotensin system: a diversity of functions and implications for CNS diseases. Pflugers Arch.

[4] Drews HJ, Klein R, Lourhmati A (2021). Losartan improves memory, neurogenesis and cell motility in transgenic Alzheimer's mice. Pharmaceuticals (Basel).

[5] Ciobica A, Bild V, Hritcu L, Padurariu M, Bild W (2011). Effects of angiotensin II receptor antagonists on anxiety and some oxidative stress markers in rat. Open Med.

[6] Chrissobolis S, Luu AN, Waldschmidt RA, Yoakum ME, D'Souza MS (2020). Targeting the renin angiotensin system for the treatment of anxiety and depression. Pharmacol Biochem Behav.

[7] Gong S, Deng F (2022). Renin-angiotensin system: the underlying mechanisms and promising therapeutical target for depression and anxiety. Front Immunol.

[8] Ahire YS, Bairagi VA, Somavanshi DB, Jadhav SR, Jadhav SB, Jagtap SD (2024). Expanding telmisartan’s therapeutic horizon: exploring its multifaceted mechanisms beyond cardiovascular disorders. Futur J Pharm Sci.

[9] Sánchez-Lemus E, Benicky J, Pavel J, Saavedra JM (2009). In vivo angiotensin II AT1 receptor blockade selectively inhibits LPS-induced innate immune response and ACTH release in rat pituitary gland. Brain Behav Immun.

[10] Lynch JJ 3rd, Castagné V, Moser PC, Mittelstadt SW (2011). Comparison of methods for the assessment of locomotor activity in rodent safety pharmacology studies. J Pharmacol Toxicol Methods.

[11] Rodgers RJ, Dalvi A (1997). Anxiety, defence and the elevated plus-maze. Neurosci Biobehav Rev.

[12] Raj PV, N MR, Nayak RP, Rao SN (2018). A study on anxiolytic activity and locomotor behavior of Curcuma amada rhizomes using Wistar albino rats. Int J Basic Clin Pharmacol.

[13] DE PB (1953). The measurement of the influence of drugs on voluntary activity in mice. Br J Pharmacol Chemother.

[14] Bhosale U, Yegnanarayan R, Prachi P, Zambare M, Somani RS (2011). Study of CNS depressant and behavioral activity of an ethanol extract of Achyranthes Aspera (Chirchita) in mouse model. Ann Neurosci.

[15] Srinivasan J, Suresh B, Ramanathan M (2003). Differential anxiolytic effect of enalapril and losartan in normotensive and renal hypertensive rats. Physiol Behav.

[16] Swetha ES, Aithal S, Rubina A (2014). Evaluation of antianxiety activity of angiotensin receptor blockers in albino mice. Sch Acad J Pharm.

[17] Saavedra JM (2012). Angiotensin II AT(1) receptor blockers as treatments for inflammatory brain disorders. Clin Sci (Lond).

[18] Villapol S, Saavedra JM (2015). Neuroprotective effects of angiotensin receptor blockers. Am J Hypertens.

[19] Olivier JD, Vinkers CH, Olivier B (2013). The role of the serotonergic and GABA system in translational approaches in drug discovery for anxiety disorders. Front Pharmacol.

[20] Cianchetti S, Del Fiorentino A, Colognato R, Di Stefano R, Franzoni F, Pedrinelli R (2008). Anti-inflammatory and anti-oxidant properties of telmisartan in cultured human umbilical vein endothelial cells. Atherosclerosis.

[21] Subramanian A (2018). Evaluation of in vitro antioxidant activity of Oxalis latifolia Kunth and its role in the treatment of neurodegenerative diseases. Eur J Pharm Med Res.

[22] Yoo SM, Choi SH, Jung MD, Lim SC, Baek SH (2015). Short-term use of telmisartan attenuates oxidation and improves Prdx2 expression more than antioxidant β-blockers in the cardiovascular systems of spontaneously hypertensive rats. Hypertens Res.

